# Improving paediatric antimicrobial stewardship in hospital-based settings: why, where and how?

**DOI:** 10.1093/jacamr/dlaa011

**Published:** 2020-03-13

**Authors:** E Tanner, A P S Munro, J Gray, H Green, M Rutter, C E Jones, S N Faust, M Alderton, S V Patel

**Affiliations:** 1 University of Southampton Medical School, University Hospital Southampton NHS Foundation Trust, Southampton, UK; 2 Department of Paediatric Immunology & Infectious Diseases, University Hospital Southampton NHS Foundation Trust, Southampton, UK; 3 NIHR Wellcome Trust Clinical Research Facility, University Hospital Southampton NHS Foundation Trust, Southampton, UK; 4 Faculty of Medicine and Institute for Life Sciences, University of Southampton, Southampton, UK; 5 Department of Paediatrics, University Hospital Southampton NHS Foundation Trust, Southampton, UK

## Abstract

**Background:**

Antimicrobial resistance (AMR) is being recognized as a priority by healthcare organizations across the world. However, many children are managed on IV antimicrobials in hospital with very little consideration of antimicrobial stewardship issues.

**Objectives:**

A nurse-led paediatric ambulatory outpatient parenteral antimicrobial therapy (OPAT) service, managing children with common infections being ambulated on short courses of IV antimicrobials, was introduced within Southampton Children’s Hospital in January 2018. We evaluated the impact of this service in terms of the quality of antimicrobial prescribing and timing of ambulation in children presenting with common infections.

**Methods:**

All cases managed within the service were reviewed in two separate 2 month time periods: prior to introduction of the service (September–October 2016) and then prospectively after its introduction (September–October 2018).

**Results:**

A total of 96% of IV antibiotic management decisions at 48 h were deemed appropriate in 2018, compared with 75% in 2016. A total of 64% of patients were ambulated on IV antibiotics at some point during their treatment course in 2018, compared with 19% in 2016. However, a significant proportion of antimicrobial decisions made at the point of presentation to hospital remained suboptimal in 2018.

**Conclusions:**

Children are commonly managed with IV antibiotics in hospital. We demonstrate marked improvements in appropriate antimicrobial use through the introduction of a nurse-led ambulatory OPAT service. In addition, such a service can promote a greater proportion of children being ambulated from hospital, freeing up valuable inpatient beds and potentially delivering cost savings that can be used to fund such services.

## Introduction

Antimicrobial resistance (AMR) is an increasing threat, recognized as a priority by healthcare organizations all over the world.^1–3^ It is understood that one primary driver of resistance to antimicrobials is antibiotic prescribing, with increased rates of inappropriate prescribing driving resistance by increasing selection pressures on bacteria.^4^ Although attention has focused on adults, there are increasing data to suggest a high burden of antimicrobial-resistant infections in young infants.^5^ One of the most important ways to reduce AMR in healthcare settings is antimicrobial stewardship, defined by NICE as a process that ‘embodies an organizational or healthcare-system-wide approach to promoting and monitoring judicious use of antimicrobials to preserve their future effectiveness’.^6^ Paediatric clinicians have an important role in ensuring appropriate and judicious antibiotic use, as up to 40% of UK hospitalized children receive antibiotics at any one time, either as inpatients or administered on an ambulatory basis.^7^ Data from the USA demonstrate that there is much room for improvement in paediatric hospital prescribing, especially in regard to inappropriate initiation of IV therapy or prolonged IV therapy when oral stepdown would have been appropriate.^8^^,^^9^ There has recently been a move towards much shorter courses of antibiotics for infections, with earlier stepdown from IV to oral being advocated in adult practice for conditions such as bone and joint infections, Gram-negative bacteraemia and endocarditis.^10–12^ A recent systematic review has highlighted where high-quality evidence supports these practices in children.^13^

However, there are conflicting clinical pressures when deciding whether children presenting with fever and infective symptoms require prompt initiation of empirical IV antibiotics. With ever more emphasis being placed on the recognition and early treatment of sepsis to reduce morbidity and death, as-yet-unpublished national data from the UK suggest that broad-spectrum antibiotic treatment in emergency departments has risen by almost 30% in the last 5 years (NHS England, unpublished data). Initiating empirical antibiotic treatment in only the sickest patients at high risk of death remains an important challenge.

At our regional children’s hospital in the UK, which serves a regional population of about 500 000 children and has approximately 9000 admissions per year, the paediatric infectious diseases and antimicrobial stewardship (PID/PAS) team has been proactive in managing children with complex infections requiring prolonged courses of IV antimicrobials at home (≥5 days following discharge from hospital) through the implementation of a tertiary paediatric outpatient parenteral antimicrobial therapy (tertiary p-OPAT) service in July 2012.^14^^,^^15^ In January 2018, this programme was expanded to include children with common infections requiring short courses of IV antimicrobials lasting <5 days (ambulatory p-OPAT) through the introduction of a nurse-led clinic where children on IV antibiotics returned to a hospital-based ambulatory unit for daily review. The nurses were trained by the PID/PAS team and were directly supported by the patient’s primary clinical team (general paediatrics or other paediatric specialities) on the suitability of children to stop or step down antibiotic therapy. The PID/PAS team had overall oversight of the service and reviewed management of all cases managed within the service. This short report describes how setting up a formalized service for this large cohort of children presenting to hospital with common infections not only provides opportunities for admission avoidance and early discharge through the delivery of safe ambulatory care, but also ensures that antimicrobial stewardship principles are adhered to.

## Methods

We evaluated the impact of our ambulatory p-OPAT service by carrying out a review of antibiotic prescribing and ambulation in two separate 2 month time periods: one carried out prior to introduction of the service (September–October 2016) and a second carried out prospectively after its introduction (September–October 2018). All children initiated on IV antibiotics for non-complex infections likely to be primarily managed in local as well as regional hospitals were included in each time period. The appropriateness of antibiotic prescribing and ambulation decisions were assessed following the completion of each episode of care by a general paediatrician (M.A.) and a consultant in paediatric infectious diseases (S.V.P.). In addition, the two assessors estimated the likely impact on rates of ambulation and antimicrobial use if optimal decisions had been made at the time of presentation to hospital (t = 0). The data were extrapolated to provide an estimate of the impact of the service over a 12 month time period. Ethical approval to conduct this service evaluation was granted by the Ethics Committee of the University of Southampton (ERGO 42242).

## Results

A total of 67 patients were identified in the retrospective group (2016) and 78 in the prospective group (2018). Table 1 outlines the pathologies managed in 2018. Ceftriaxone was the most commonly used empirical IV antibiotic during both periods (66% of cases in 2016 and 91% in 2018). In 2018, 96% of IV antibiotic management decisions made at 48 h were deemed appropriate, compared with 75% in 2016, the most common reason being failure to stop IV antibiotics at 48 h when no longer indicated. No adverse events occurred after cessation of antibiotics in either patient cohort. In 2018, 50 (64%) of all paediatric presentations/admissions started on IV antibiotics for non-complex infections were ambulated at some point during their treatment course, compared with only 13 (19%) in 2016, despite the opportunity for children to return to the acute paediatric ward for daily IV antibiotics existing in 2016. Although the total number of IV antibiotic days was similar in both time periods (205 in 2016 and 201 in 2018), inpatient days on IV antibiotics dropped from 172 in 2016 to 106 in 2018.

**Table 1.** Pathologies managed with IV antibiotics (2018)

Working diagnosis	Patients, *n* (%)
Query sepsis	15 (19)
LRTI	13 (16)
Pyelonephritis/upper UTI	9 (12)
Cellulitis	8 (10)
Fever without known source	7 (9)
URTI	7 (9)
Lymphadenitis	3 (4)
Meningitis	2 (3)
Scalded skin syndrome	2 (3)
Gastroenteritis	2 (3)
Infected eczema	2 (3)
Seizure	2 (3)
Scarlet fever	1 (1)
Glomerulonephritis	1 (1)
Subcutaneous abscess	1 (1)
Query endocarditis	1 (1)
Rash	1 (1)
Conjunctivitis	1 (1)

UTI, urinary tract infection; LRTI, lower respiratory tract infection; URTI, upper respiratory tract infection.

However, despite the ambulatory OPAT service having a significant impact on decision-making at 48 h, a significant proportion of antibiotic-prescribing decisions made at the time of presentation to hospital (t = 0 decisions) could have been improved in 2018. Twenty-one (27%) children started on IV antibiotics were deemed to have been managed suboptimally, with no antimicrobial therapy required in nine (43%) and oral antibiotics being indicated in nine (43%). The main presentation for which antimicrobials were commenced inappropriately was lower respiratory tract infection; of the 13 children managed with IV antibiotics for lower respiratory tract infections, antibiotic therapy was not indicated in 6 (46%) and oral antibiotics rather than IV antibiotics would have been more appropriate in 3 (23%). In terms of ambulation, although a far greater proportion of children were ambulated on IV antibiotics at some point during their admission in 2018 compared with 2016, there was almost no change in the proportion being immediately ambulated (admission avoidance): 35% in 2016 compared with 43% in 2018. Evaluation of the impact of optimized t = 0 decision-making suggested a 23% reduction in courses of total IV antibiotics, 33% increase in children being ambulated on IV antibiotics without admission (admission avoidance) and a 15% reduction of inpatient bed days for children on IV antibiotics (Table 2).

**Table 2.** Potential impact of improved decision-making at the time of presentation to hospital (t = 0 decisions) on antimicrobial use and admissions

	2018 data	Estimated impact of optimized decision-making	Extrapolation over 12 months	Total activity over 12 months
Admission avoidance	21	28 (↑33%)	41 extra admissions avoided	164 patients
Inpatient bed days	106	90 (↓15%)	93 extra bed days saved	524 bed days
Potential IVAb courses avoided	78	60 (↓23%)	104 courses of IVAbs avoided	346 IVAb courses administered

IVAb, IV antibiotics; ↑, increase of; ↓, decrease of.

## Discussion

This report describes the management of a cohort of children with common infections within a nurse-led ambulatory antibiotic service. This differs from the model of care currently offered in most hospitals, in which children being ambulated on IV antibiotics return daily for administration of their medication outside of a formalized antimicrobial service. The range of pathologies managed within our service is representative of those routinely seen in local hospitals. We have demonstrated that introducing a dedicated cohort of nurses to deliver this service, trained and supported by infection experts, has proven effective in improving the quality of antibiotic prescribing and encouraging timely ambulation of children from hospital. The increase in ambulation is likely to reflect clinician confidence in the service. However, there is room for improvement; although the service has resulted in a marked improvement in decision-making at 48 h, it has had less of an impact on decisions made when a child presents to hospital (t = 0 decisions). If t = 0 antibiotic-prescribing decision-making were to be optimized, significant benefits could be realized in terms of reducing unnecessary exposure to antibiotics as well as reducing rates of admission to hospital.

Focusing on antimicrobial prescribing in children is especially timely because of the recent emphasis being placed on sepsis. This message has emerged at a time when rates of invasive bacterial infections in children are extremely low, due to the introduction of highly effective vaccines against pathogens such as *Streptococcus pneumoniae* (pneumococcus), *Neisseria meningitidis* (meningococcus) and *Haemophilus influenzae* type b (Hib).^16^^,^^17^ Unfortunately this focus on sepsis, along with high-profile medicolegal cases, has resulted in clinicians becoming increasingly risk averse when managing children presenting with infection. This is likely to have contributed to the almost 30% rise in broad-spectrum antibiotic treatment in UK emergency departments in the last 5 years (NHS England, unpublished data). Aligning approaches to sepsis and antimicrobial stewardship needs to be a priority, especially in children.^18^^,^^19^

### Limitations

One of the major limitations of this study is the relatively small number of patient episodes observed over a short period of time. Another of the limitations is the location in which this study was conducted; we recognize that the resources and personnel available in a tertiary children’s hospital differ significantly from those available in local hospitals. Although there are a number of barriers to the successful implementation of ambulatory p-OPAT services in local hospital settings, including a lack of funding and lack of formalized training opportunities for healthcare professionals to perform this role, we think that many of these can be overcome through the development of regional infection networks, in which PID/PAS teams within tertiary children’s hospitals train and support general paediatricians, clinical pharmacists, microbiologists and nurses working in local hospitals. Funding could be obtained through savings made from admission avoidance and reduced length of inpatient stays. To address the current knowledge gap, evidence-based UK and Ireland good practice recommendations, focusing on antimicrobial stewardship and ambulation of children presenting to hospital with common infections, are being drafted by BSAC and the Royal College of Paediatrics and Child Health (RCPCH). These will support clinicians in local hospitals to shorten the duration of antibiotic courses, encourage earlier stepdown from IV to oral therapy and facilitate timely ambulation from hospital when appropriate.

### Conclusions

We hope that the success of our paediatric ambulatory p-OPAT service, which has relied on collaboration between nurses, general paediatricians, paediatric specialists and tertiary infectious diseases specialists, will prompt other tertiary children’s hospitals to implement similar services. More importantly, we hope that it will encourage PAS teams in regional children’s hospitals to support their colleagues working in local hospitals. This could be achieved by introducing inreach or outreach educational programmes, collaborating on regional antimicrobial guidelines and setting up regional PAS networks. Only by doing this can we ensure high-quality antimicrobial prescribing adhering to the principles of antimicrobial stewardship in all hospital settings, reducing the emergence of AMR in children.

## Funding

This study was conducted as part of a University of Southampton BMedSci project and as part of routine clinical work.

## Transparency declarations

None to declare.
